# Revisiting Snodgrass and Vanderwart in photograph form: The Keele Photo Stimulus Set (KPSS)

**DOI:** 10.3758/s13428-024-02351-1

**Published:** 2024-02-08

**Authors:** Jamie Adams, Susan M. Sherman, Helen L. Williams

**Affiliations:** School of Psychology, Dorothy Hodgkin Building, Keele University, Keele, Staffordshire, ST5 5BG UK

**Keywords:** Photographic stimuli, Snodgrass and Vanderwart, Object recognition

## Abstract

Over the last 40 years, object recognition studies have moved from using simple line drawings, to more detailed illustrations, to more ecologically valid photographic representations. Researchers now have access to various stimuli sets, however, existing sets lack the ability to independently manipulate item format, as the concepts depicted are unique to the set they derive from. To enable such comparisons, Rossion and Pourtois (2004) revisited Snodgrass and Vanderwart’s (1980) line drawings and digitally re-drew the objects, adding texture and shading. In the current study, we took this further and created a set of stimuli that showcase the same objects in photographic form. We selected six photographs of each object (three color/three grayscale) and collected normative data and RTs. Naming accuracy and agreement was high for all photographs and appeared to steadily increase with format distinctiveness. In contrast to previous data patterns for drawings, naming agreement (*H* values) did not differ between grey and color photographs, nor did familiarity ratings. However, grey photographs received significantly lower mental imagery agreement and visual complexity scores than color photographs. This suggests that, in comparison to drawings, the ecological nature of photographs may facilitate deeper critical evaluation of whether they offer a good match to a mental representation. Color may therefore play a more vital role in photographs than in drawings, aiding participants in judging the match with their mental representation. This new photographic stimulus set and corresponding normative data provide valuable materials for a wide range of experimental studies of object recognition.

Perhaps the most commonly used picture stimuli in cognitive research are the line drawings published by Snodgrass and Vanderwart (1980). To date, this set of stimuli has been cited over 7000 times (Google Scholar, January 2024). Consisting of 260 unique items, the pictures consist of simple outlines of common, everyday objects (e.g., apple, shoe) presented in black ink. A range of normative data accompanies the pictures, providing researchers with a pool of standardized stimuli that can be filtered according to a range of attributes, including: i) semantic category, e.g., animals, furniture, fruit; ii) indices of naming agreement – to what extent participants agree when providing a label for the picture; iii) mental imagery agreement – how well the picture aligns with mentally generated representations of the object; iv) visual complexity – how much detail is present in the picture, and v) familiarity – how frequently the object is experienced in everyday life. The stimuli have often been revised since initial publication; for example, the set has been expanded with additional items (Cycowicz et al., 1997); standardized for child samples (Berman et al., 1989; Cycowicz et al., 1997); acquired culturally appropriate normative data (e.g., Spanish: Sanfeliu & Fernandez, 1996; Chinese: Yoon et al., 2004; and Russian: Tsaparina et al., 2011); and bolstered with additional data, e.g., testing the relationship between reaction time and naming agreement (Székely et al., 2003). Similar line-drawn object picture sets have also been published (e.g., Dell’Acqua et al., 2000).

Despite their widespread use as research tools, line drawings have been criticized for their relative simplicity and lack of realism (e.g., Viggiano et al., 2004). While some theories of object recognition appear to support the use of object outlines, identifying shape as the factor most important for successful recognition (e.g., recognition-by-components theory; Biederman, 1987), others suggest surface details such as color and texture play an equally important role in forming object representations (Tanaka et al., 2001; Tarr & Bulthoff, 1998). As such, many researchers now favor more detailed picture stimuli. To this end, Rossion and Pourtois (2004) revisited the Snodgrass and Vanderwart (1980) line drawings and digitally re-drew the same objects, adding additional surface texture and shading details. They also provided greyscale and color versions of all items (as opposed to the greyscale-only items found in the Snodgrass and Vanderwart, 1980 set; see Fig. 1 for example items). This revision now appears to be favored over the original Snodgrass and Vanderwart (1980) set by many cognitive researchers (Ensor et al., 2019; Rollins & Riggins, 2018; Stenberg, 2006; Wolk et al., 2008) – almost certainly attributable to the increased detail and ability to choose whether color has an influence on results.

**Fig. 1:**
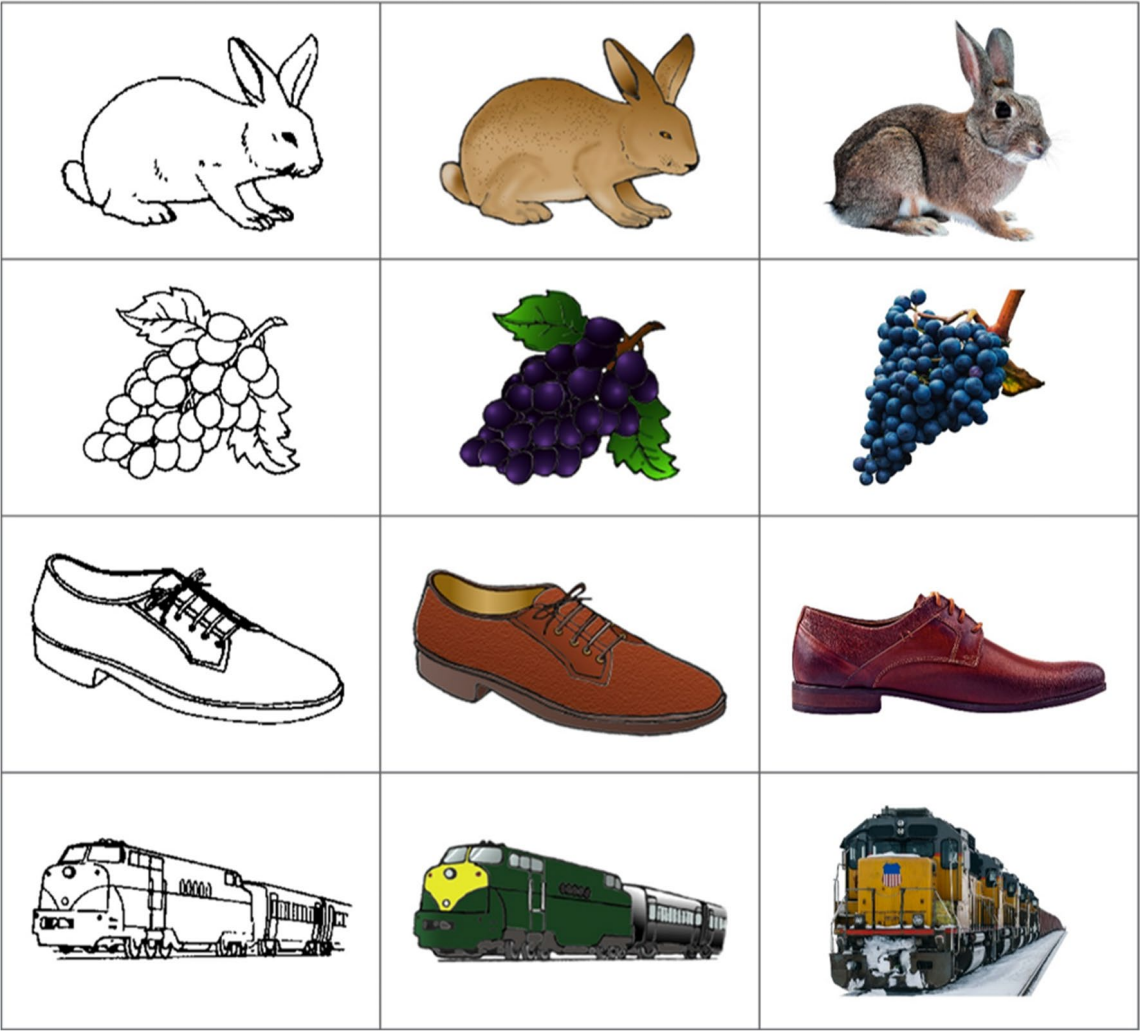
Example images: *left*: line drawings from Snodgrass and Vanderwart (1980); *middle*: matched revisions of the same items with texture and color additions from Rossion and Pourtois (2004); *right*: matched color photographs sourced for the current study (greyscale versions were created from the color versions)

Our aim was to create a new set of photograph stimuli, in which the same objects found in the color/shaded drawings of Rossion and Pourtois (2004) were similarly depicted in another format. We were interested in this from an episodic recognition memory standpoint. There is evidence to suggest that the physical characteristics of stimuli play an important role in memorability (Ensor et al., 2019), and thus the creation of matched photographs is important so that recognition for the same objects/concepts can be systematically compared across different *levels* of stimuli detail; for example: i) words, i.e., written item labels; ii) greyscale shaded drawings; iii) color drawings; iv) black-and-white photographs, v) color photographs. The Picture Superiority Effect (PSE) is the highly robust and replicable phenomenon whereby memory is better for pictures compared to words. The effect is present in children, adolescents, and older adults (Ally et al., 2008; Whitehouse et al., 2006), and has generally been shown to manifest as both increased recollection and familiarity in recognition memory paradigms (Dewhurst & Conway, 1994; Rajaram, 1993, 1996; Wagner et al., 1997; Yonelinas, 2002). Certain methodological decisions might have an impact on the emergence of the PSE in recognition memory judgments. One such decision is the type of picture stimuli used, yet there is often very little justification or appraisal of alternative formats in the literature. The extent to which successful picture recognition may be affected by different types of picture (e.g., those with varying degrees of detail) is unknown, despite there being little consistency across studies with regard to the types of visual items used.

According to perceptual distinctiveness accounts of the PSE, stimulus sets containing a high level of item-to-item variability (i.e., greater physical differences between one stimulus and the next) are more likely to be recognized than those with low variability, as they serve to highlight the uniquely individuating characteristics of each item, which in turn increases the likelihood of retrieval (Mintzer & Snodgrass, 1999; Nelson et al., 1976). Across the vast field of recognition memory literature, a wide range of formats have been used when presenting to-be-remembered stimuli to participants; although the PSE itself is a robust phenomenon, when some results are obtained in response to drawings and others are obtained in response to photographs this inconsistency can make it difficult to reconcile more fine-grained differential effects. One such example concerns the contributions of recollection and familiarity processes to recognition of words versus pictures in memory-impaired populations such as patients with mild cognitive impairment (e.g., Ally & Budson, 2007; Ally et al., 2008, 2009; Embree et al., 2012; Sanger & Anderson, 2022). By creating a new set of object photographs – carefully selected to resemble existing illustrated representations – the impact of stimuli format can be independently examined, since the specific objects/concepts are the same. To reduce experimenter bias and ensure the new set of photographs consisted only of items that closely resembled the Rossion and Pourtois (2004) depictions, normative data were collected for three distinct variations of each object (see Fig. 2) and used to select the best match. The specific data collected for each item replicated the methodology of Rossion and Pourtois (2004), with measures of naming agreement, mental imagery agreement, familiarity, visual complexity, and color diagnosticity obtained.

**Fig. 2:**
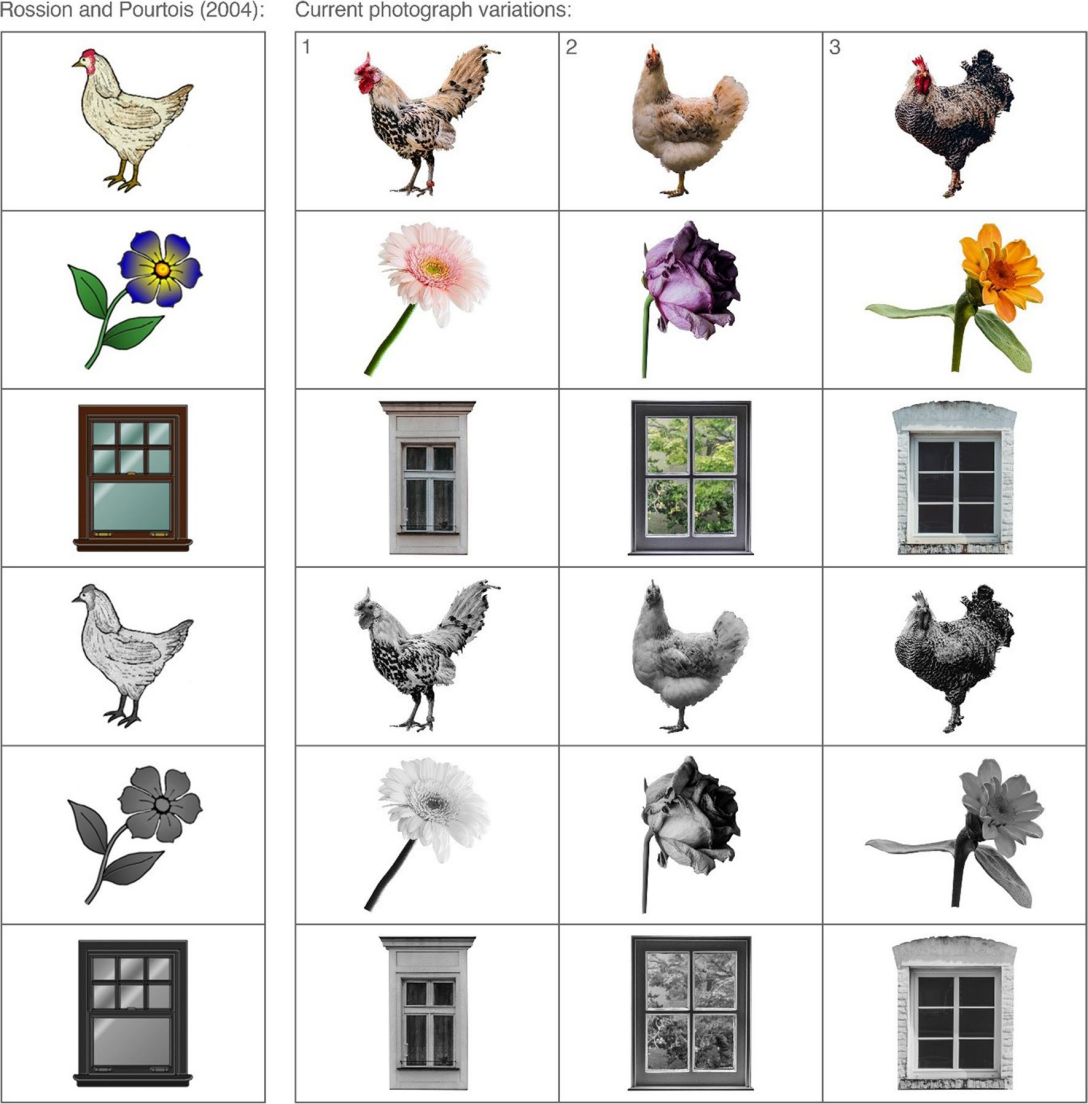
Example drawings (greyscale and color) from Rossion and Pourtois (2004) alongside the three possible photograph variations that were chosen to similarly depict the object in the current study. Normative data were collected for all photographs and used to determine which of the three variations best depicted the intended concept

## Method

### Participants

In total, 377 participants completed the online experiment (see Table 1 for demographic information). In line with Rossion and Pourtois (2004), this sample size provided 20 data points for each of the five response types. Participants were recruited from Prolific (where they received payment at the rate of £5/h) and via the Keele School of Psychology research participation system (where they received course credit). The experiment took approximately 25 min to complete. All participants were required to be aged between 18 and 59 years (obtained range 18–59 years). As the experiment involved typing the English labels for a range of image stimuli, participants were asked whether English was their first language; all but one participant indicated that English was indeed their first language (99.2%).

**Table 1:** Gender, mean age (and standard deviation) of participant sample

Gender	*N*	Age
Female	196	33.22 (11.28)
Male	171	33.15 (10.3)
Non-binary	2	23.5 (4.95)
Unspecified	5	29.4 (6.11)
Total	377	33.16 (10.84)

### Stimuli

Photographs were obtained online with the aim of similarly depicting the same everyday objects as those found in a subset of 136 of Rossion and Pourtois’s (2004) line drawings. We selected this subset to suit the needs of the recognition memory experiments we were planning to conduct using these stimuli. Our inclusion/exclusion criteria were: we included images depicting objects where the object name was between 4 and 7 letters in length; excluded multiple-word names (e.g., French horn, frying pan, spool of thread, etc.); excluded what we considered to be American English words (e.g., wrench) that would have lower familiarity for our participants from the UK; and excluded items where a photograph of the image would not be able to be found that closely matched the original line drawings (e.g., “star”).

In seeking photographs to match the drawings, the inherent subjectivity involved in this process may have led to images that were not a reliable ‘match’ to the concepts they were selected with the aim of depicting (e.g., the photograph chosen to depict the concept “bottle” may inadvertently provoke the majority of participants to give the label “wine”). To address this issue and ensure all photographs more objectively depicted the same concepts as the original drawings, three different photograph variations were found for each object with the aim of identifying the best ‘match’. Emphasis was placed on variety across the three variations, with at least one photograph selected to closely resemble the line-drawn depiction and another selected to offer a more modern depiction.

Photograph stimuli were obtained by searching open-source, copyright-free image websites (e.g., www.unsplash.com; www.pexels.com) for photographs that depicted the same everyday objects as the shaded drawings (see osf.io/8ft9z/ for the full list of image references). The matching process produced a total of 408 unique photographs (three variations for each of the 136 objects). Each image was imported into Adobe Photoshop (20.0.04 Release) where the background was removed to isolate the object of interest from other potentially distracting visual details. This was completed manually using the magnetic lasso and polygonal lasso tools (edges were either feathered by 1 px or left un-feathered). Where appropriate, the orientation of isolated objects was adjusted to ensure they matched as closely as possible with their line-drawn counterpart (e.g., all photograph variations of the item “boot” were adjusted so the toe was facing left and the heel facing right, as in the shaded drawing); this was often achieved by flipping or mirroring the object to ‘correct’ the direction.

**Fig. 3:**
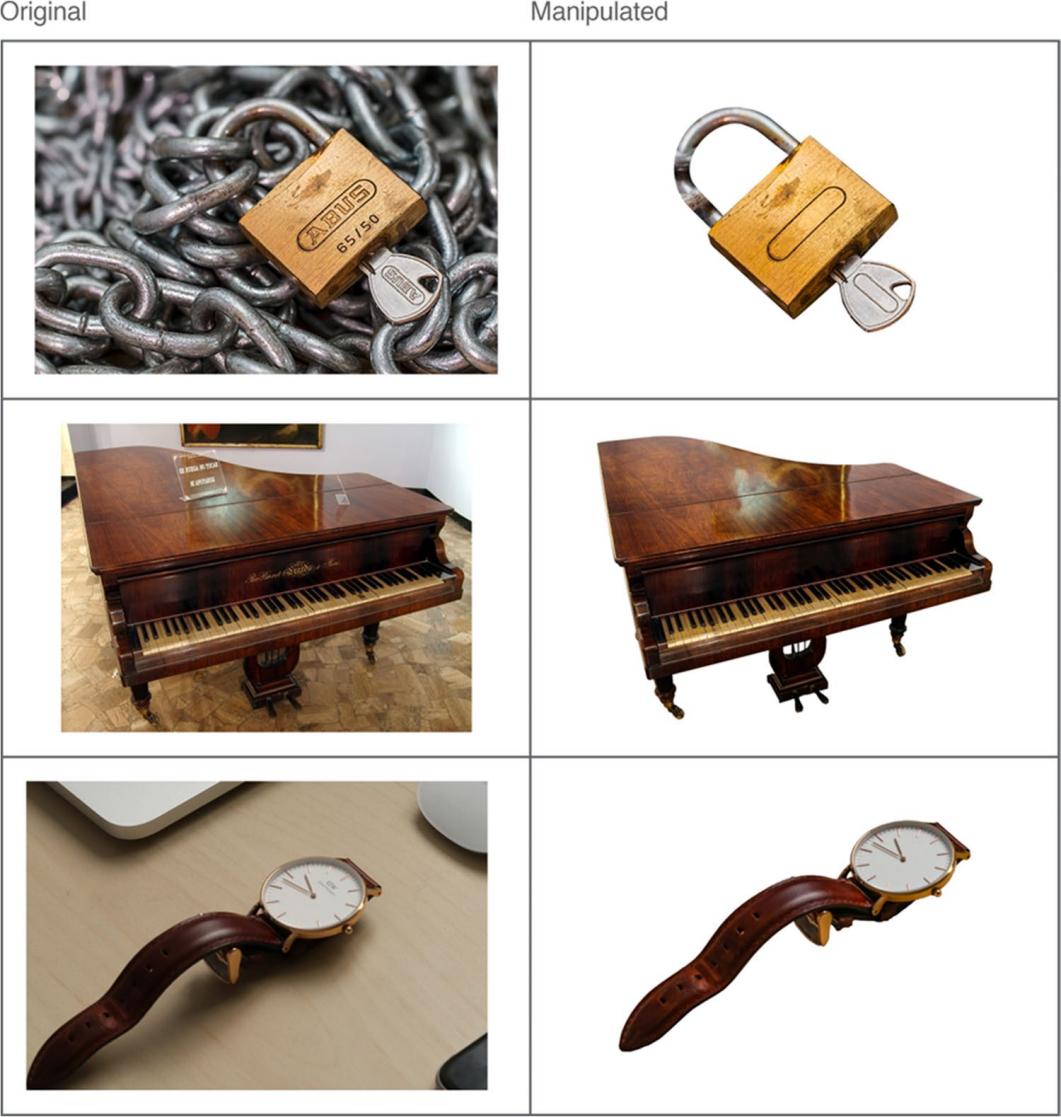
Examples of text (and background) removal from photographs

Despite isolating objects from their background, a small number of photographs still contained irrelevant and potentially distracting details. For example, in one photograph variation of the item “piano” there was a sign on the object that may have impacted how the item was named or rated. Such details were removed as best as possible using the clone stamp and content-aware fill tools. Any obvious text (e.g., brand names) and numbers were also removed from photographs using similar methods (see Fig. 3). The primary aim of the current experiment was to obtain photographs that could be clearly distinguished as a unique stimulus format among words and shaded drawings; it is conceivable that combining these formats (i.e., inadvertently including photographs that also contain written words) might affect recognition performance in ways that are not directly comparable to items defined only by a single category. Any text was therefore removed, apart from a couple of exceptions where such details were integral to the depiction of the object (e.g., the numbers found on a ruler or a clock face).

All photographs were exported from Photoshop in .PNG format in their original color, and in greyscale by setting saturation levels to 0. Final edits were completed in Adobe Lightroom (Classic, 8.2 Release); these were: exposure (brightness) adjustments for images that appeared too light or too dark; highlight reduction when some areas of the object were too bright compared to the rest of the photograph; shadow raising when some areas of the object were too dark compared to the rest of the photograph; and noise reduction was applied whenever the aforementioned edits had introduced unwanted noise/grain artefacts.

The changes made to each image were systematically applied to both the color and greyscale versions (e.g., if one variation of “shoe” had an exposure increase of .010 for the color version, the greyscale version also received an exposure increase of .010). Some color-specific adjustments were made to the color photographs only, however; common artefacts such as chromatic aberration (purple fringing) were corrected and white balance was normalized. Finally, all photographs were placed on a 600 × 600 pixel white background, with the isolated objects resized to fill this frame as much as possible; i.e., some items were constrained by height, whilst others were constrained by width (stimuli available from osf.io/8ft9z/).

### Design

This was a descriptive experiment, and a mix of quantitative and qualitative data were gathered. All participants completed three blocks: i) Naming/Familiarity; ii) Visual Complexity/Color Diagnosticity; iii) Mental Imagery Agreement. Presentation order of blocks was counterbalanced across participants. All responses were provided on a five-point scale, apart from the Naming task, which consisted of a single-word typed response. In the Naming/Familiarity and Visual Complexity/Color Diagnosticity blocks, participants were required to provide two response types to each unique item shown. The first two blocks each contained 45 items, while the third block contained 46 items. In total, each participant responded to all 136 unique objects, half presented in greyscale and half in color.

*Procedure*

Ethical approval was obtained from Keele University’s Psychology Research Ethics Committee. The procedure was similar to that of Rossion and Pourtois (2004) except for the following: Rossion and Pourtois (2004) collected each response type (Naming, Familiarity, etc.) from independent samples, with participants responding to every object; the current study instead used a within-subjects design where all participants saw all 136 unique objects but only provided each type of response for a subset of objects. Rossion and Pourtois (2004) collected data from participants in a classroom setting (tested individually or in groups); in the current study participants completed the tasks online in their own environment. Rossion and Pourtois (2004) presented their stimuli on either a computer (Naming, Mental Imagery Agreement) or projected onto a large screen (Familiarity, Visual Complexity); in the current study, participants viewed the stimuli on their own computers (the software enforced the use of full screen). In the Naming task, Rossion and Pourtois (2004) used a microphone to record responses whereas in the current study participants typed their response using the keyboard. In the Familiarity task, Rossion and Pourtois (2004) only presented their stimuli for 3 s, whereas in the current study image presentation time was self-paced as the Naming and Familiarity tasks were shown on the same screen. While Rossion and Pourtois (2004) used physical data sheets for participants’ rating scale responses, participants in the current study used the mouse to click a response button. The experimental definitions and instructions used in the current study (detailed below) were matched as closely as possible to those used by Rossion and Pourtois (2004).

Data collection was conducted via Qualtrics for collection of consent, demographics, and computer compatibility data; and Pavlovia (Peirce et al., 2019) for the experimental tasks. Within each block, items were presented individually, in random order; once participants had provided the required response(s), a fixation cross was presented for a 1-s interstimulus interval before the next item was shown.

In the Naming/Familiarity block, participants were first asked “What is the name of the item depicted?” Participants were instructed to name each photograph as briefly and unambiguously as possible, with one word only, by typing their answer into the response box. If they did not know the name of an item or had a tip-of-the-tongue experience, participants were instructed to type “no” for their answer. The term “don’t know” was avoided, so as not to encourage participants to deviate from single-word responses. Following the naming judgment, with the same photograph still present on screen, participants were asked “How familiar is the item depicted?”. Participants were instructed to judge each photograph according to how usual or unusual the item was in their realm of experience; specifically, familiarity was defined as “the degree to which you come in contact with or think about the concept” to encourage participants to rate the concept itself, rather than the particular way it was depicted. Participants were shown a five-point scale (ranging from 1 = *very unfamiliar* to 5 = *very familiar*) and were encouraged to use the full range of the scale across the set of photographs.

In the Visual Complexity and Color Diagnosticity block, participants were asked “How visually complex is this picture?” and responded using a five-point scale (1 = *very simple* to 5 = *very complex*). Complexity was defined to participants as “the amount of detail in the picture”; in contrast to the familiarity ratings, participants were encouraged here to rate the complexity of the picture itself, rather than the real-life item. If the photograph shown was greyscale, participants simply moved on to the next item. If the item shown was in color, however, participants were also required to make a color diagnosticity judgment. This concept was defined as “how typical/normal the color of the item is,” instructing participants to rate on a five-point scale (from 1 = *Not at all diagnostic*, i.e., this item could be in any other color equally well; to 5 = *Highly diagnostic*, i.e., this item appears only in this color in real life). Participants were instructed to utilize the full range of options on the scale when making visual complexity and color diagnosticity judgments.

Due to the slight change in procedure and increased task complexity compared to the other blocks, Mental Imagery Agreement ratings were always collected in a separate block (i.e., not alongside any other response types). After a 1-s fixation period, participants were presented with a written label for 3 s (e.g., “cat”) and told to focus their attention on the word. Once the written word disappeared, a beep tone was played alongside the instruction “close your eyes and imagine this item” (participants were encouraged to close their eyes and begin imagining the item as soon as they heard the tone, but the written instructions were included as a further prompt). After 3 s, a second beep tone sounded to alert participants to open their eyes, where they were presented with a photograph of the item they had been instructed to imagine. Participants were asked to “rate the agreement between your mental image and the picture” (1 = *low agreement*, 5 = *high agreement*). The degree of agreement was defined as “how similar your mental image of the item is to the picture shown.” For all tasks, responses were self-paced; the timing was only controlled during the study/imagine section of the Mental Imagery Agreement block. Full details of all instructions are available from osf.io/8ft9z/.

### Data processing

Naming responses for each photograph were manually assessed for spelling and typing errors. Most errors were unambiguous and easy to correct (e.g., “anker” = “anchor,” “peguin” = “penguin,” “ssnowman” = “snowman”) or consisted of transforming plural words to singular (or vice versa; e.g., “sock” to “socks”)^1^. There were instances where participants provided a sensible and correctly spelled English word, but upon examination of the photograph they were in response to, it was clear these were simply a typo and were therefore corrected (e.g., “dock” in response to a photograph of a duck, “frock” in response to a frog, and “beer” in response to a “bear”).

Though participants were instructed to only give a single word label for each image, some multiple-word responses (without spaces) were provided. On these occasions, a judgment was made regarding whether multiple words were retained or whether the response could be shortened into a single word. A general rule was applied whereby if the other words provided additional information, they were retained (e.g., “maledear” – presumably “male deer” – was kept as a two-word answer). Multiple-word responses were shortened into a single word when the intended label for the item was present and no information was lost in the process (e.g., “haircomb” was shortened to the intended answer “comb”). It is noted that there was some inherent subjectivity in this process, though as such items were not common, their overall effects are estimated to be negligible.

Finally, some responses were changed to “no” as they were clearly intended to signify that the participant did not know the name of the item; for example, “none” and “idk” (common abbreviation for “I don’t know”). This process yielded data that could be used to determine which photograph variation best matched the intended concepts (e.g., 100% of participants labeled one photograph “bottle,” indicating a perfect match) from those which did not (e.g., only 50% of participants labeled another photograph “bottle,” while the other 50% gave the label “wine,” indicating a poor match). For a full list of name adjustments see osf.io/8ft9z/.

### Analysis preparation

In preparation for analysis, mean ratings were calculated for familiarity, visual complexity, color diagnosticity, and mental imagery agreement. For naming responses, accuracy was defined as the proportion of participants reporting the correct/intended label for any given item (e.g., 80% of participants correctly labeled the photograph “moon-1” as “moon”). Percentage agreement was also calculated (i.e., the proportion of participants providing the most frequent name, regardless of whether it matched the correct/intended label) in order to compute *H* values for each item. The *H* statistic takes into account the total number of unique labels given for an item; this is especially useful for comparing similar items, as it captures information not provided by simple agreement proportions. For instance, if both variations of the photograph Moon (“moon-1” and “moon-2”) demonstrated 90% naming agreement among participants, it would appear as if they offer the same level of agreement among participants. However, “moon-1” may have received a total of two unique names (e.g., “moon,” “planet”), while “moon-2” may have received four unique names (e.g., “moon,” “planet,” “earth,” “comet”). *H* values utilize this information to determine which item shows the best naming agreement (in other words, the photograph with the least number of unique names). The original formula by Snodgrass and Vanderwart (1980) was used to calculate *H* values; see Equation 1. A *H* value of 0 indicates perfect naming agreement (all participants responded with the same label for that image). Items showing a *H* value of 1 signify two unique names were provided, with identical proportions (e.g., ten participants responded “moon” and ten participants responded “planet”). As the *H* value increases, overall naming agreement decreases. The maximum value of *H* achievable in this study would have been 4.32; to get this value, an item would have had to have been given 20 unique names.

**Equation **1 Formula for calculation of H Values (Snodgrass & Vanderwart, 1980)

1 $$H=\sum_{i=1}^{k}{p}_{i}{log}_{2}\frac{1}{{p}_{i}},$$

Mean reaction times (RTs) were also calculated for each photograph/response variable, including naming responses. For naming responses, RTs were the length of time from page onset to when participants first pressed a key on the keyboard. Participants did not have to click the input box to start typing, so the key press was the first thing they did. For all other DVs, RTs were the length of time from page onset to when participants selected a rating response with the mouse. Participants were not given any instructions regarding measurement of response times.

## Results

Summary statistics (mean and SD) for each of the measured variables are shown in Table 2. Data for our greyscale and color photographs are presented alongside existing normative values from Rossion and Pourtois (2004; including revised norms for Snodgrass and Vanderwart’s 1980 original line drawings). Data from these previous studies were not used in any statistical analyses. To examine differences across our greyscale and color photographs, independent samples *t* tests were run on the mean rating/score for each variable, as well as on their corresponding reaction times (excluding scores of color diagnosticity, which were obtained only in response to the color items and thus cannot be compared). For individual photograph scores and ratings, see osf.io/8ft9z/).

**Table 2:** Means (and standard deviations) for each of the measured variables

	Rossion & Pourtois (2004)			Current study	
Variable	S&V (1980) line drawings	Grey shaded drawings	Color shaded drawings	Grey photos	Color photos
Naming Accuracy	88.2 (17.1)	89.2 (17.2)	90.3 (16.9)	94.44 (8.18)	94.76 (7.74)
Naming Agreement (H)	0.44 (0.56)	0.38 (0.52)	0.32 (0.46)	0.24 (0.33)	0.22 (0.31)
Mental Imagery Agreement	3.73 (0.48)	3.76 (0.55)	3.74 (0.63)	3.46 (0.56)	3.74 (0.65)
Familiarity	3.59 (0.94)	3.52 (1.01)	3.44 (1.01)	4.13 (0.56)	4.19 (0.54)
Visual complexity	2.76 (1.03)	2.88 (1.03)	2.7 (0.94)	2.87 (0.62)	3.16 (0.63)
Color diagnosticity	- -	- -	3.18 (1.23)	- -	3.22 (0.84)

### Naming

Naming accuracy was very high for all photographs (*M* = 0.95), indicating that overall, the selected items closely depicted the intended concepts. Compared with the previous stimuli formats, there appears to be a steady increase in accuracy as items become more distinctive (see Table 2). Accuracy rates did not differ between the grey (*M* = 0.94) and color (*M* = 0.95) versions of the photographs, (745.64) = 0.56, *p* = .576. *H* values were also low across all items (overall *M* = 0.23; perfect agreement would be 0, maximum disagreement would be 4.32), showing that participants generally agreed on how the items should be named. Similar to naming accuracy, naming agreement also appears to steadily increase as items become more distinctive (as indicated by decreasing *H* values; see Table 2). While Rossion and Pourtois (2004) observed significantly better naming agreement for their color compared to greyscale images, for the current set of photographs *H* values did not differ between the grey (*M* = 0.24) and color (*M* = 0.22) images, *t*(743.66) = 0.62, *p* = 0.54.

A mean reaction time (RT) of 3.9 s was observed for naming responses; there was no significant difference in naming latency for grey images (*M* = 4.00s) and color images (*M* = 3.80s), (651.86) = 1.57, *p* = .117. Overall, these analyses suggest the current photographs closely resemble the drawings they were designed to match, with high levels of naming accuracy and agreement among participants. The absence of any color differences indicates there were no naming advantages when photographs were intended to be more distinctive through the addition of color.

### Mental imagery agreement

Scores of mental imagery agreement were moderate across all items (*M* = 3.60) but grey photographs (*M* = 3.46) received significantly lower mental imagery agreement scores than color photographs (*M* = 3.74), (800.06) = 6.54,*p* < .001. In addition, RTs for rating mental imagery agreement were significantly faster for color photographs (*M* = 2.81) compared to grey photographs (*M* = 3.04), (571.37) = 2.14, *p* = .033.

### Familiarity

Familiarity ratings were high overall (*M* = 4.16), with no significant difference in mean familiarity for grey (*M* = 4.13) and color (*M* = 4.19) photographs, *t*(813.19) = 1.63, *p* = .103. RTs for familiarity ratings for grey (*M* = 0.97) and color (*M* = 0.98) images also did not differ significantly, *t*(783.66) = 0.30, *p* = .762.

### Visual complexity

Visual complexity ratings were moderate across all photographs (*M* = 3.30), but color photographs (*M* = 3.16) received significantly higher visual complexity scores than grey photographs (*M* = 2.87), *t*(813.51) = 6.65, *p* < .001. RTs for ratings of visual complexity did not differ significantly across grey (*M* = 3.26) and color (*M* = 3.35) images, *t*(754.08) = 1.21, *p* = .228.

### Selection of best match stimuli

For each concept represented in the photographs, the normative naming data was assessed to establish which version (e.g., “shoe-1”, “shoe-2”, or “shoe-3”) best matched the existing line-drawn depictions of the concepts (Rossion & Pourtois, 2004). Our priority for this selection was based on identifying the images that would be most useful in studies examining recognition memory. Thus, naming was favored over the other variables as, if an item was found to primarily convey a different concept than was intended during the naming task (e.g., if a photograph of the fruit ‘orange’ was labeled ‘grapefruit’ by the majority of participants), then it would not be comparable to its line-drawn, or written-word, counterparts effectively during recognition studies. At least 20 unique naming responses were collected for each of the 816 photographs (408 grey items and 408 color items). The proportion of correct responses (i.e., names that were congruent with the intended concept) and the proportion of “don’t know” responses were calculated for each item. Photographs were excluded if they:

1. received a high proportion of “don’t know” responses (> 20%; all of the photographs depicted common, everyday objects so if a number of participants were unable to name the item, that particular photograph was considered to be a poor representation of the item);
2. were incorrectly named by the majority of participants (correct responses ≤ 50%, since it was important for the photographs to depict the same concepts as found in the shaded drawings);
3. had particularly poor naming agreement (≤ 20% participants named the object similarly). Images may not have been flagged by the second criteria (e.g., if it received four different names, each with a 25% ratio), but could still be considered poor representations of the intended concepts.

In total, 54 photographs met at least one of these criteria and were excluded. Regardless of whether these items were grey or color, it was also necessary to remove their grey or color partner since both versions would be needed to make comparisons across stimuli in a recognition experiment. Thus, a total of 64 items (32 grey/32 color) were excluded at this stage.

Next, the proportion of correct responses were compared between grey and color photographs in order to identify items showing the least difference. In order to manipulate color in recognition experiments it is important to select items where naming is congruent across color/grey images. This is because it is difficult to attribute particular recognition response patterns to the addition of color if the greyscale image of an item cannot be identified (or encoded) similarly. Variations exhibiting the least difference between color and grey images (for the proportion of correct responses) were selected as the best match, while the rest were excluded. In a number of instances, multiple variations for the same object had the same difference score. For example, all three photographs of the item “balloon” exhibited perfect naming agreement, irrespective of whether they were presented in color or grey. For items where more than one variation remained, independent subjective rankings were carried out by two of the authors (JA and HW) to determine which variation best depicted the intended concept. Items where there was agreement as to which variation best depicted the intended concept were selected for inclusion in the final stimuli list (ranking details available from osf.io/8ft9z/). For any items where there was disagreement between researchers’ rankings, one of the variations was selected at random.

## Discussion

For naming responses (accuracy, agreement [*H*], and RTs), no differences were observed between the grey and color photographs. This was expected for accuracy and agreement scores; the addition/absence of color should not alter how participants identify (and thus label) items, except in rare instances whereby a lack of color may lead to the misidentification of an object (e.g., incorrectly labelling a greyscale photograph of an orange as “grapefruit”). The data indicates, however, that this was not common, with the greyscale photographs exhibiting equally high levels of naming accuracy as the color photographs. The absence of RT differences between the color and greyscale sets was not expected for naming responses. It is reasonable to assume that color photographs – with an additional layer of contextual information compared to grey photographs – might be identified (and therefore named) quicker than grey images (e.g., a color photograph of an orange should avoid the potential ambiguity that might accompany a greyscale depiction, which could be confused for another type of fruit). Indeed, Rossion and Pourtois (2004) demonstrated RTs consistent with this hypothesis, with color drawings showing significantly quicker RTs than greyscale drawings. The lack of difference in the current data could be attributable to ceiling effects, i.e., all photographs that were sufficiently unambiguous were quickly identified irrespective of whether they were presented in greyscale or color. Examination of the other naming data, showing similarly high levels of accuracy and agreement across grey and color, supports this.

Scores of mental imagery agreement produced particularly interesting results between the grey and color items. Grey photographs exhibited a significantly poorer match with participants imagined presentation of the objects than the color items. Color differences were not observed previously with drawings (Rossion & Pourtois, 2004). Comparison of our current data with that obtained in earlier studies demonstrates how greyscale photographs show uniquely lower mental imagery agreement scores compared with any of the other stimuli formats (see Table 2). To imagine the objects, it seems likely that participants would conjure an image of how they naturally see the item in their everyday lives, which for the majority of participants, would presumably be a color representation. Therefore, when presented with greyscale depictions, participants appear to have considered the image to not align quite as well as those presented in color. However, it is unclear why a similar pattern is not also evident when comparing grey and color drawings (Rossion & Pourtois, 2004). It may be that photographs promote stricter internal criteria when participants must decide whether an item is a good match to their mental image. With line-drawn/illustrated items, participants may simply accept that the items are baseline depictions that are only able to match their real-world mental images to a certain degree, thus leading to a generally more liberal response bias throughout. The addition of color to a drawing may therefore do very little to further reconcile the match between the drawing and real-world mental representation. When participants are responding to photographs, however, the ecological nature of the items may facilitate deeper critical evaluation of whether they offer a good match to a mental representation and thus promote a more conservative response bias. Color may therefore be a far more important factor in photographs than it is in drawings for allowing participants to decide whether an item matches well with their mental representation.

There were no differences in familiarity scores across grey and color photographs. This result was expected – participants were asked to rate the degree to which they came in contact with or think about the concept itself rather than the particular depiction shown and there is no reason why color should influence such ratings. Visual complexity, on the other hand, where participants were required to directly rate the amount of detail in the image, did show an expected difference. Color photographs were rated as significantly more visually complex than grey items, presumably due to their additional layer of information. When compared to the data obtained for drawings by Rossion and Pourtois (2004), the greyscale photographs showed comparable levels of visual complexity, while the color photographs showed higher levels than any of the other formats. It is unclear why the photographs in the current experiment showed color differences, when grey and color drawings did not differ, though it may tie in with the hypothesis proposed to explain the mental imagery agreement data. Participants may apply stricter internal criteria when rating stimuli that are perceived as being closer to how they would be experienced in real life, i.e., when viewing a color photograph of a rabbit, it is difficult to see how the item could be made any more visually complex than it already is (at least in a 2D medium). It is probable that participants notice the absence of color when viewing the greyscale items, since they depict the items in a way that they are not usually seen, thus determine that these items could be made more complex if they were shown in color and so give lower visual complexity ratings as a result.

One theoretical question in the object recognition literature that this experiment cannot answer is: what do photographs truly add to recognition relative to line drawings (e.g., Biederman, 1987; Singer et al., 2023; Uttl et al., 2006)? This is not a question that this study was designed to address as we did not directly compare Snodgrass and Vanderwart’s (1980) line drawings or Rossion and Pourtois’s (2004) textured drawings against our new greyscale and color photographic stimuli. The objective of the current study was to establish a new set of ecological photograph stimuli that can facilitate comparison of image stimulus types. As the KPSS depicts the same concepts as earlier image sets, and images have been matched against those to a high degree, this set can be compared against the original line drawings of Snodgrass and Vanderwart (1980) and the color/greyscale drawings of Rossion & Pourtois (2004) in future research to examine the effects of additional detail and stimulus format on recognition performance.

The photograph stimuli created in this study may prove useful for a range of cognitive researchers, and not only those looking to utilize a set of high-quality and realistic object images within recognition memory research. First, this is the only known stimulus set where the same items are matched across many distinct levels of both detail and color. Second, the range of normative data collected for each unique photograph (408 color items + 408 greyscale items) allows for additional flexibility given items can be filtered according to color, naming agreement, familiarity, etc. The provision of three images per item also allows researchers to use different photographs to represent the same item (e.g., as matched lures in an alternative forced-choice recognition paradigm). While other photograph stimulus sets include a greater number of alternates per object (e.g., Migo et al., 2013), the KPSS is the only set, to our knowledge, that was specifically designed to both match the original Snodgrass and Vanderwart (1980) depictions and include color as well as greyscale alternates.

## Footnotes

1 Some responses were a little more ambiguous and necessitated comparison to the photographs they were in response to for additional clarity (e.g., a photograph depicting a plug that would fit into North American electrical sockets was labeled as “usplug”; given that some of the participants were from the UK, it seemed likely this particular participant was reporting the item to be a “U.S. (i.e., United States) plug,” as opposed to a British plug.

## References

[CR1] Ally BA (2012). Using pictures and words to understand recognition memory deterioration in amnestic mild cognitive impairment and Alzheimer’s disease: A review. Current Neurology and Neuroscience Reports.

[CR2] Ally BA, Budson AE (2007). The worth of pictures: Using high density event-related potentials to understand the memorial power of pictures and the dynamics of recognition memory. NeuroImage.

[CR3] Ally BA, Gold CA, Budson AE (2009). The picture superiority effect in patients with Alzheimer’s disease and mild cognitive impairment. Neuropsychologia.

[CR4] Ally BA, Waring JD, Beth EH, McKeever JD, Milberg WP, Budson AE (2008). Aging memory for pictures: Using high-density event-related potentials to understand the effect of aging on the picture superiority effect. Neuropsychologia.

[CR5] Berman S, Friedman D, Hamberger M, Snodgrass JG (1989). Developmental picture norms: Relationships between name agreement, familiarity, and visual complexity for child and adult ratings of two sets of line drawings. Behavior Research Methods, Instruments, & Computers.

[CR6] Biederman I (1987). Recognition-by-components: A theory of human image understanding. Psychological Review.

[CR7] Cycowicz YM, Friedman D, Rothstein M, Snodgrass JG (1997). Picture naming by young children: norms for name agreement, familiarity, and visual complexity. Journal of Experimental Child Psychology.

[CR8] Dell’Acqua R, Lotto L, Job R (2000). Naming times and standardized norms for the Italian PD/DPSS set of 266 pictures: Direct comparisons with American, English, French, and Spanish published databases. Behavior Research Methods, Instruments, & Computers.

[CR9] Dewhurst, S. A., & Conway, M. A. (1994). Pictures, images, and recollective experience. *Journal of Experimental Psychology: Learning, Memory, and Cognition*, *20*(5), 1088–1098. https://psycnet.apa.org/doi/10.1037/0278-7393.20.5.1088 10.1037//0278-7393.20.5.1088 7931096

[CR10] Embree LM, Budson AE, Ally BA (2012). Memorial familiarity remains intact for pictures but not for words in patients with amnestic mild cognitive impairment. Neuropsychologia.

[CR11] Ensor TM, Surprenant AM, Neath I (2019). Increasing word distinctiveness eliminates the picture superiority effect in recognition: Evidence for the physical-distinctiveness account. Memory & Cognition.

[CR12] Migo EM, Montaldi D, Mayes AR (2013). A visual object stimulus database with standardized similarity information. Behavior Research Methods.

[CR13] Mintzer MZ, Snodgrass JG (1999). The Picture Superiority Effect: Support for the distinctiveness model. The American Journal of Psychology.

[CR14] Nelson DL, Reed VS, Walling JR (1976). Pictorial Superiority Effect. Journal of Experimental Psychology: Human Learning and Memory.

[CR15] Peirce, J., Gray, J. R., Simpson, S., MacAskill, M., Höchenberger, R., Sogo, H., … Lindeløv, J. K. (2019). PsychoPy2: Experiments in Behavior Made Easy. *Behavior Research Methods*, *51*(1), 195–203. 10.3758/s13428-018-01193-y 10.3758/s13428-018-01193-y PMC6420413 30734206

[CR16] Rajaram S (1993). Remembering and knowing: two means of access to the personal past. Memory & Cognition.

[CR17] Rajaram S (1996). Perceptual effects on remembering: Recollective processes in picture recognition memory. Journal of Experimental Psychology: Learning, Memory, and Cognition.

[CR18] Rollins, L., & Riggins, T. (2018). Age-related differences in subjective recollection: ERP studies of encoding and retrieval. *Developmental Science,**21*(3), e12583. 10.1111/desc.12583 10.1111/desc.12583 28677331

[CR19] Rossion, B., & Pourtois, G. (2004). Revisiting Snodgrass and Vanderwart’s object pictorial set: the role of surface detail in basic-level object recognition. *Perception*, *33*(2), 217–236. 10.1068/p5117 10.1068/p5117 15109163

[CR20] Sanfeliu MC, Fernandez A (1996). A Set of 254 Snodgrass-Vanderwart pictures standardized for Spanish: Norms for name agreement, image agreement, familiarity, and visual complexity. Behavior Research Methods, Instruments, & Computers.

[CR21] Sanger BD, Anderson ND (2022). Familiarity deficits for words and objects in amnestic mild cognitive impairment in a context minimizing the role of recollection. The Journals of Gerontology: Series B.

[CR22] Singer JJ, Cichy RM, Hebart MN (2023). The spatiotemporal neural dynamics of object recognition for natural images and line drawings. Journal of Neuroscience.

[CR23] Snodgrass JG, Vanderwart M (1980). A standardized set of 260 pictures: Norms for name agreement, image agreement, familiarity, and visual complexity. Journal of Experimental Psychology: Human Learning and Memory.

[CR24] Stenberg G (2006). Conceptual and perceptual factors in the picture superiority effect. European Journal of Cognitive Psychology.

[CR25] Székely, A., D’Amico, S., Devescovi, A., Federmeier, K., Herron, D., Iyer, G., … Bates, E. (2003). Timed picture naming: Extended norms and validation against previous studies. *Behavior Research Methods, Instruments, & Computers*, *35*(4), 621–633. 10.3758/BF03195542 10.3758/bf03195542 14748507

[CR26] Tanaka J, Weiskopf D, Willliams P (2001). The role of color in high-level vision. Trends in Cognitive Sciences.

[CR27] Tarr MJ, Bülthoff HH (1998). Image-based object recognition in man, monkey and machine. Cognition.

[CR28] Tsaparina D, Bonin P, Méot A (2011). Russian norms for name agreement, image agreement for the colorized version of the Snodgrass and Vanderwart pictures and age of acquisition, conceptual familiarity, and imageability scores for modal object names. Behavior Research Methods.

[CR29] Uttl B, Graf P, Santacruz P (2006). Object color effects identification and repetition priming. Scandinavian Journal of Psychology.

[CR30] Viggiano MP, Vannucci M, Righi S (2004). A new standardized set of ecological pictures for experimental and clinical research on visual object processing. Cortex.

[CR31] Wagner AD, Gabrieli JDE, Verfaellie M (1997). Dissociations between familiarity processes in explicit recognition and implicit perceptual memory. Journal of Experimental Psychology: Learning, Memory, and Cognition.

[CR32] Whitehouse AJO, Maybery MT, Durkin K (2006). The development of the picture-superiority effect. British Journal of Developmental Psychology.

[CR33] Wolk DA, Signoff ED, DeKosky ST (2008). Recollection and familiarity in amnestic mild cognitive impairment: a global decline in recognition memory. Neuropsychologia.

[CR34] Yonelinas AP (2002). The nature of recollection and familiarity: a review of 30 years of research. Journal of Memory and Language.

[CR35] Yoon, C., Feinberg, F., Luo, T., Hedden, T., Gutchess, A. H., Chen, H.-Y. M., … Park, D. C. (2004). A cross-culturally standardized set of pictures for younger and older adults: American and Chinese norms for name agreement, concept agreement, and familiarity. *Behavior Research Methods, Instruments, & Computers*, *36* (4), 639–649. 10.3758/BF03206545 10.3758/bf03206545 15641410

